# Rab3B enhances the stabilization of DDX6 to promote lung adenocarcinoma aggressiveness

**DOI:** 10.1186/s10020-024-00848-1

**Published:** 2024-06-04

**Authors:** Guodong Yao, Shan Yu, Feng Hou, Zunyu Xiao, Guangqi Li, Xiaobin Ji, Jigang Wang

**Affiliations:** 1 Department of Pathology, Affiliated Hospital of Qingdao University, Qingdao, 266071 Shandong Province China; 2 Department of Pathology, The Second Affiliated Hospital of Harbin Medical University, Harbin, Heilongjiang Province China; 3 Department of Imaging, The Fourth Affiliated Hospital of Harbin Medical University, Harbin, Heilongjiang Province China

**Keywords:** DDX6, Lung adenocarcinoma, Rab3B, Stability, Tumorigenesis

## Abstract

**Background:**

Liver kinase B1 (*LKB1*) is frequently mutated in lung adenocarcinoma, and its loss contributes to tumor progression.

**Methods:**

To identify LKB1 downstream genes that promote lung adenocarcinoma aggressiveness, we performed bioinformatical analysis using publicly available datasets.

**Results:**

Rab3B was upregulated in *LKB1*-depleted lung adenocarcinoma cells and suppressed by LKB1 overexpression. CREB protein was enriched at the promoter of *Rab3B* in lung cancer cells. Silencing of *CREB* abrogated the upregulation of *Rab3B* upon *LKB1* loss. Immunohistochemistry revealed the elevated expression of Rab3B in lung adenocarcinomas relative to adjacent normal tissues. Upregulation of Rab3B was significantly associated with lymph node metastasis, advanced tumor stage, and reduced overall survival in lung adenocarcinoma patients. Knockdown of *Rab3B* suppressed and overexpression of Rab3B promoted the proliferation, colony formation, and migration of lung adenocarcinoma cells in vitro. In a mouse xenograft model, *Rab3B* depletion restrained and Rab3B overexpression augmented the growth of lung adenocarcinoma tumors. Mechanistically, Rab3B interacted with DDX6 and enhanced its protein stability. Ectopic expression of DDX6 significantly promoted the proliferation, colony formation, and migration of lung adenocarcinoma cells. *DDX6* knockdown phenocopied the effects of *Rab3B* depletion on lung adenocarcinoma cells. Additionally, DDX6 overexpression partially rescued the aggressive phenotype of *Rab3B*-depleted lung adenocarcinoma cells.

**Conclusion:**

*LKB1* deficiency promotes Rab3B upregulation via a CREB-dependent manner. Rab3B interacts with and stabilizes DDX6 protein to accelerate lung adenocarcinoma progression. The Rab3B-DDX6 axis may be potential therapeutic target for lung adenocarcinoma.

## Introduction

Non-small cell lung cancer (NSCLC) is a common malignant disease and the leading cause of cancer-related deaths worldwide (Desai et al. 2023). Lung adenocarcinoma is the major histological subtype of NSCLC (Vokes et al. 2022). Many tumour suppressors and oncogenes are mutated in NSCLC and play critical roles in NSCLC development and progression (Skoulidis and Heymach 2019; Tan and Tan 2022). Of these, liver kinase B1 (*LKB1*, also called *STK11*) is attracting much attention as its mutation rate is as high as approximately 20% in NSCLC (Shackelford et al. 2013). LKB1 is known to function as a tumor suppressor (Li et al. 2021). Loss of LKB1 has been found to accelerate lung cancer tumorigenesis and metastasis (Ji et al. 2007). The tumor-promoting effects elicited by LKB1 loss are related to alteration of the transcriptional and epigenetic programs of cancer cells (Pierce et al. 2021; Hollstein et al. 2019; Kottakis et al. 2016). Pierce et al. (2021) reported that loss of LKB1 can modulate chromatin accessibility to activate SOX17, which in turn drives a second wave of epigenetic changes and enhances lung adenocarcinoma metastasis. Hence, identification of LKB1 downstream genes is of significance for understanding the mechanisms governing lung cancer progression.

Rab3B is a member of the Rab family of Ras-related monomeric GTPases and has the capacity to regulate vesicular traffic (Tsetsenis et al. 2011; Rotondo et al. 2009). It has been documented that knockdown of Rab3B blocks calcium-dependent exocytosis in rat anterior pituitary cells (Lledo et al. 1993). In addition to neuronal/secretory cells, Rab3B is also found in cancer cells (Tsunedomi et al. 2022; Liu et al. 2014). Tsunedomi et al. (2022) reported that Rab3B is upregulated in hepatocellular carcinoma. In glioma, Rab3B is also upregulated and has a prognostic significance (Liu et al. 2014). Knockdown of Rab3B leads to reduced proliferation and invasion in breast cancer cells (Ye et al. 2014). In addition, Rab3B upregulation contributes to prostate cancer cell survival (Tan et al. 2012). Despite these findings, it remains unclear whether Rab3B could modulate lung cancer cell behaviors.

DDX6 belongs to the DEAD-box RNA helicase family and participates in a variety of biological processes such as RNA metabolism, translation initiation, and pre-mRNA splicing (Smillie and Sommerville 2002). DDX6 can be recruited to the CCR4-NOT complex to repress mRNA translation (Mathys et al. 2014). Di Stefano et al. (2019) also demonstrated that DDX6 is required for the translational suppression of target mRNAs in P-bodies of stem cells. In several cancer types including gastric cancer, colorectal cancer, and glioblastoma (Taniguchi et al. 2018; Nakagawa et al. 1999; Cho et al. 2016), DDX6 is found to play an oncogenic role.

In an attempt to identify key genes involved in lung cancer progression, we performed bioinformatical analysis using publicly available Gene Expression Omnibus (GEO) datasets. We show that Rab3B is differentially expressed between lung adenocarcinoma and adjacent normal tissues and induced upon LKB1 deficiency. We also reveal an interaction between Rab3B and DDX6, which contribute to the aggressive phenotype in lung adenocarcinoma cells both in vitro and in vivo.

## Materials and methods

### Cell culture

Two NSCLC cell lines (H1299 and H1792) carrying wild-type *LKB1* and 3 *LKB1*-mutated NSCLC cell lines (A549, H460, and H1944) were obtained from the American Type Culture Collection (ATCC, Manassas, VA, USA). The cell lines were cultured in RPMI-1640 medium (Sigma-Aldrich, St. Louis, MO, USA) supplemented with 10% fetal bovine serum (FBS) and 1% penicillin–streptomycin (Sigma-Aldrich).

### Tissue samples and immunohistochemistry (IHC)

Primary tumor and adjacent normal tissue samples were obtained from a total of 92 patients diagnosed with lung adenocarcinoma. These patients underwent surgery between January 2014 and July 2015. None of them received preoperative anti-cancer treatments. The surgically resected tissue samples were processed for IHC. IHC was performed using anti-Rab3B antibody (1:50 dilution; Cell Signaling Technology, Danvers, MA, USA). Immunostained slides were scored by two independent pathologists who were blinded to patient information. H-score (range 0–300) was calculated using the following formula (Conde et al. 2019): 3 × percentage of strongly stained cells + 2 × percentage of moderately stained cells + 1 × percentage of weakly stained cells. A Rab3B H-score cutoff of 50 was used to differentiate high or low Rab3B expression.

### DNA constructs, siRNAs, and cell transfections

The cDNA sequences of *LKB1*, *Rab3B*, and *DDX6* were cloned to the pcDNA3.1( +) vector. For generation of Flag-tagged Rab3B constructs, the *Rab3B* cDNA sequence was ligated into the pFLAG-CMV-1 vector. Short hairpin RNAs (shRNAs) targeting *Rab3B* and *DDX6* were cloned into the pLKO.1-Puro lentiviral vector: shRab3B#1, AGTGCAAAGGAGAACATCAGT, and shRab3B#2, CAGGGACTTAGAGAACAGTCT; shDDX6#1, CAACACAATCAATAATGGCAC, and shDDX6#2, GGATGTGACCTCCACAAAAGG. Two small interfering RNAs (siRNAs) targeting *LKB1* and *CREB* were synthesized: siLKB1#1, AAGGAAAUUCAACUACUGA, and siLKB1#2, UCCUCAAGAAGAAGAAGUU; siCREB#1, GAGAGAGGUCCGUCUAAUG, and siCREB#2, GCUGGCUAACAAUGGUACC.

DNA constructs were transfected using Lipofectamine 3000 transfection reagent (Thermo Fisher Scientific, San Jose, CA, USA). siRNAs were transfected at a final concentration of 30 nM using Lipofectamine RNAiMAX transfection agent (ThermoFisher Scientific). shRNAs were transfected into 293 T cells together with lentiviral packaging vectors to produce shRNA-expressing lentivirus. Lentivirus-infected cells were cultured in 2 μg/mL puromycin (Sigma-Aldrich) to select stable knockdown cells.

### Quantitative real-time PCR (qRT-PCR) analysis

Total RNA was isolated using TRIzol (Thermo Fisher Scientific) and reversely transcribed using the SuperScript VILO cDNA Synthesis Kit (Thermo Fisher Scientific) as per the manufacturer’s protocol. qRT-PCR was performed using the SYBR Green PCR master mix (Thermo Fisher Scientific). The qRT-PCR primers are listed as below: *Rab3B* forward 5′-GGTTGTTCCCACTGAGAAGG-3′, *Rab3B* reverse 5′-TTGCACTGGCTTCAAAGAAA-3′; *LKB1* forward 5′-TCTACACTCAGGACTTCACG-3′, *LKB1* reverse 5′-GTTCATACACACGGCCTT-3′; *DDX6* forward, 5′-GGCTGGGAAAAGCCATCT-3′, and *DDX6* reverse, 5′-ACCTGATCTTCCAATACG-3′; *GAPDH* forward, 5′-CCACCCATGGCAAATTCCATGGCA-3′, *GAPDH* reverse, 5′-TCTAGACGGCAGGTCAGGTCCACC-3′. All mRNA levels were normalized to *GAPDH* mRNA.

### Chromatin immunoprecipitation (ChIP) assay

After treatment, lung cancer cells were fixed with formaldehyde and lysed in ChIP lysis buffer. Cross-linked chromatin were sheared by sonication to fragments of 300–500 base pairs. The chromatin fragments were incubated with anti-CREB antibody (Cell Signaling Technology) or control IgG. The immunocomplexes were captured by protein G beads, and DNA was extracted. The DNA fragments were quantified by quantitative PCR using the following primers: 5′-AGTAGAGACAGGGTTTCACCA-3′ and 5′-ACACAAGCCCGAGCAACCCTT-3′.

### Western blotting, co-immunoprecipitation, and mass spectrometry

Whole cell extracts were prepared using radioimmunoprecipitation assay (RIPA) buffer (Cell Signaling Technology) supplemented with protease inhibitors (Cell Signaling Technology). Protein samples were separated in sodium dodecyl sulfate–polyacrylamide gel electrophoresis (SDS-PAGE) and transferred onto nitrocellulose membranes. The membranes were incubated with the primary antibodies against LKB1 (Cell Signaling Technology), Rab3B (Thermo Fisher Scientific), DDX6 (Cell Signaling Technology), CREB (Cell Signaling Technology), Flag tag (Thermo Fisher Scientific) and GAPDH (Cell Signaling Technology) at 4 °C overnight. Then, the membranes were incubated with horseradish peroxidase-conjugated secondary antibodies. The blot signals were visualized using enhanced chemiluminescence reagents (Cell Signaling Technology).

For co-immunoprecipitation assay, cells were lysed in ice-cold RIPA buffer containing protease inhibitors and incubated with anti-Flag tag, anti-Rab3B, anti-DDX6, or IgG overnight at 4 °C. The antibody-containing protein complexes were captured by protein A/G agarose beads and resolved by SDS-PAGE. Silver-stained gels of immunoprecipitated proteins were subjected to trypsin digestion and mass spectrometry analysis.

### DDX6 protein stability analysis

A549 and H1944 cells were transfected with Rab3B-targeting shRNAs or control shRNAs and treated with 100 μg/ml cycloheximide (Sigma-Aldrich) for 0, 1, 2, and 4 h. Cells were lysed and protein lysates were analyzed by Western blotting to determine the levels of endogenous DDX6 protein. To inhibit proteasome-dependent protein degradation, cells were treated with 5 µM MG132 (Selleck, Houston, Texas, USA) for 2, 6, and 10 h before Western blot analysis.

### Cell proliferation assay

Cells were seeded (1000 cells per well) in 96-well plates and cultured for 0–5 days. The cell number was determined at indicated time points, and cell growth curves were accordingly plotted.

### Colony formation assay

Lung cancer cells transfected with indicated constructs were seeded (400 cells per well) in 6-well plates and cultured for 10–14 days. Colonies were stained with crystal violet solution (Sigma-Aldrich) and counted.

### Transwell migration assay

Lung cancer cells transfected with indicated constructs were suspended in serum-free media and plated on Transwell inserts (8-μm pore diameter). The medium containing 10% FBS was added to the lower chamber. After incubation for 24 h, the cells on the lower surface of the inserts were stained with crystal violet and counted under a microscope.

### In vivo tumorigenic studies

Lung cancer cells stably transfected with indicated constructs were injected subcutaneously into 4–6-week-old male Balb/c nude mice (4 × 10^6^ cells per mouse). Tumor sizes were measured with a caliper every 5 days. Mice were sacrificed 25 days after cell injection, and the xenograft tumors were resected and photographed.

### Bioinformatic analysis

The transcriptomic data were extracted from GEO datasets GSE32863, GSE51266, GSE75037, and GSE133715. Differentially expressed genes were analyzed using the GEO2R online tool (https://www.ncbi.nlm.nih.gov/geo/geo2r/) for microarray data and the R Bioconductor package DESeq2 for RNA sequencing data. Genes with an adjusted *p*-value < 0.05 were considered as significantly differentially expressed genes. The web-based tool, Kaplan–Meier plotter (https://kmplot.com/analysis/) (Lánczky and Győrffy 2021), was employed to determine the relationship between RAB3B expression and overall survival of lung adenocarcinoma patients using public RNA sequencing datasets.

### Statistics

Data are expressed as mean ± standard deviation and were analyzed for significance with the Student’s *t*-test or one-way analysis of variance followed by Tukey’s post-hoc test. The relationship between Rab3B expression and patient overall survival was analyzed by the Kaplan–Meier method, and statistical significance was determined with the Log-rank test. The association of Rab3B expression with clinicopathologic parameters of lung adenocarcinoma patients was studied using the chi-square test. A *P* value < 0.05 was considered to be statistically significant.

## Results

### Rab3B expression is associated with lung adenocarcinoma prognosis

**Fig. 1:**
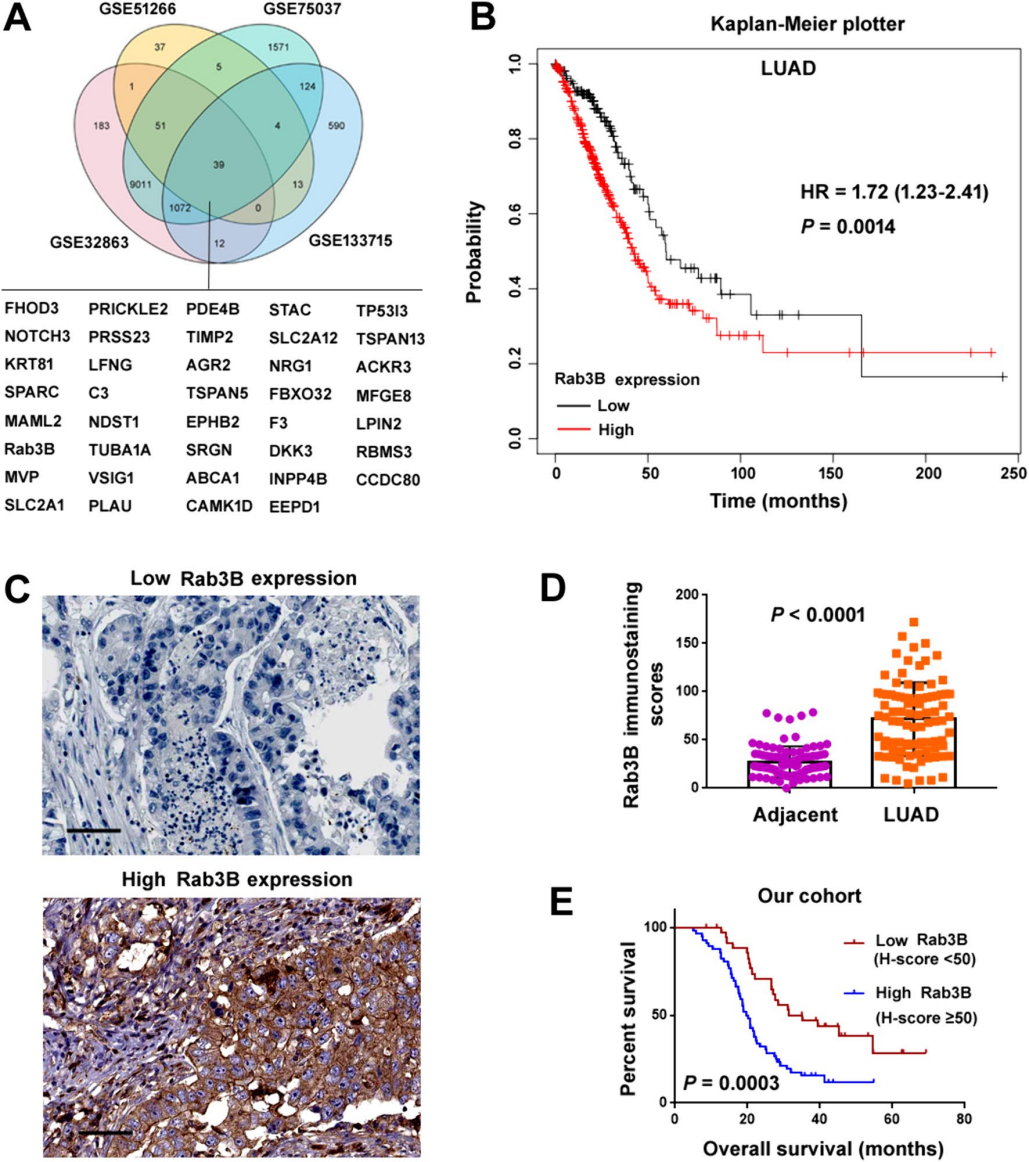
Rab3B expression is associated with lung adenocarcinoma prognosis. **A** Venn diagram showing a list of differentially expressed genes that are common to the 4 GEO datasets. **B** Overall survival curves of high versus low expression of Rab3B in lung adenocarcinoma (LUAD) based on Kaplan–Meier plotter databases. **C**, **D** Immunohistochemistry for Rab3B in paired tumor and adjacent normal tissues from a cohort of 92 lung adenocarcinoma patients. **C** Representative images of lung adenocarcinoma specimens with low and high Rab3B expression. Scale bar: 100 μm. **D** Rab3B H-scores were compared between LUAD and adjacent normal tissues. **E** Overall survival curves of high versus low expression of Rab3B according H-scores in our cohort of LUAD patients

We initially sought to identify the key genes that can be regulated in response to LKB1 loss and involved in lung adenocarcinoma progression. Transcriptomic data from public GEO datasets GSE32863, GSE51266, GSE75037, and GSE133715 were analyzed. Differentially expressed genes between lung adenocarcinomas and non-malignant adjacent tissues (GSE32863 and GSE75037) and between LKB1 loss and reexpression in lung adenocarcinoma cells (GSE51266 and GSE133715) were determined. As a result, we obtained a list of 39 differentially expressed genes that were common to the 4 GEO datasets analyzed (Fig. 1A). Among them, Rab3B mRNA expression was significantly associated with overall survival of lung adenocarcinoma patients (*P* = 0.0014; Fig. 1B), which was determined based on Kaplan–Meier plotter. To validate the clinical significance of Rab3B in lung cancer, immunohistochemistry for Rab3B was performed in paired tumor and adjacent normal tissues from a cohort of 92 lung adenocarcinoma patients. We found that Rab3B H-scores were significantly higher in lung adenocarcinoma than those in adjacent lung tissues (Fig. 1C and D). All the lung adenocarcinoma cases tested were divided into low and high Rab3B subgroups based on the H-score cutoff of 50. Rab3B expression was significantly correlated with lymph node metastasis (*P* = 0.0014) and tumor stage (*P* = 0.0058), but not with age, sex, or primary tumor size (Table 1). Most importantly, lung adenocarcinoma patients with high Rab3B expression in tumors had significantly shorter overall survival than those with low Rab3B expression (*P* = 0.0003; Fig. 1E). Taken together, high Rab3B expression is associated with poor prognosis of lung adenocarcinoma.

**Table 1:** The relationship between Rab3B expression and clinicopathological characteristics of lung adenocarcinoma patients

Characteristics	Rab3B expression (H-score cutoff = 50)		*P* value
	Low (n = 36)	High (n = 56)	
Age, years			0.8085
≤ 60	17	25	
> 60	19	31	
Sex			0.7605
Female	8	14	
Male	28	42	
Primary tumor size, cm			0.2131
≤ 5	24	30	
> 5	12	26	
Lymph node metastasis			0.0014
Yes	9	33	
No	27	23	
Stage			0.0058
I/II	26	24	
III/IV	10	32	

### Loss of LKB1 induces Rab3B expression via CREB-dependent transcription

**Fig. 2:**
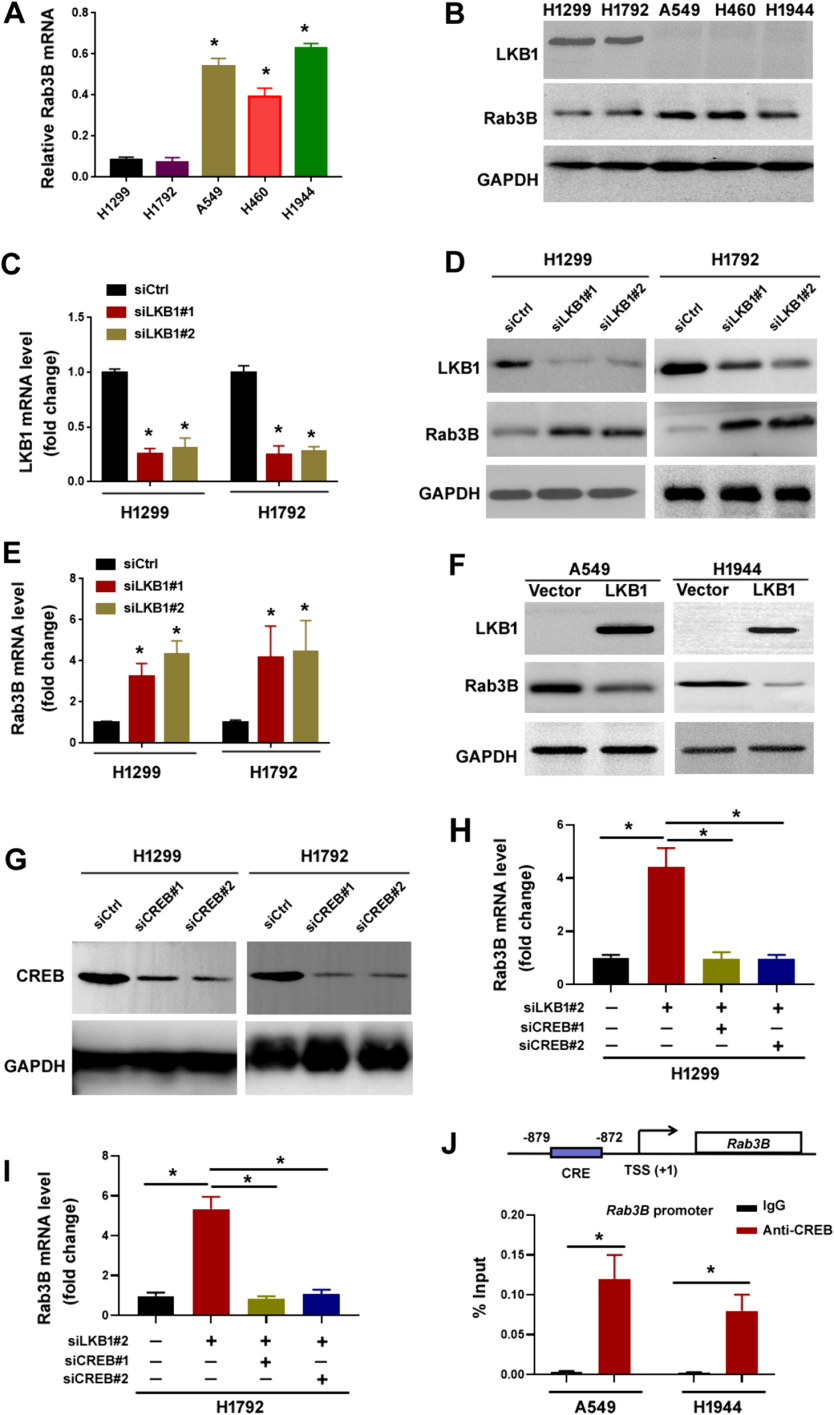
CREB is required for Rab3B upregulation in NSCLC cells in response to *LKB1* deficiency. **A**, **B** Analysis of Rab3B mRNA **A** and protein **B** levels in different NSCLC cell lines. ^*^P < 0.05 in (**A**). **C** Analysis of LKB1 mRNA levels in H1299 and H1792 cells transfected with indicated siRNAs. ^*^P < 0.05. **D** Western blot analysis of LKB1 and Rab3B protein levels in H1299 and H1792 cells transfected with indicated siRNAs. **E** Analysis of Rab3B mRNA levels in H1299 and H1792 cells transfected with indicated siRNAs. ^*^P < 0.05. **F** Western blot analysis of LKB1 and Rab3B protein levels in A549 and H1944 cells transfected with indicated constructs. **G** Western blot analysis of CREB protein levels in H1299 and H1792 cells transfected with indicated siRNAs. **H**, **I** Analysis of Rab3B mRNA levels in H1299 and H1792 cells transfected with indicated siRNAs. ^*^P < 0.05. **J** *Top*, a schematic illustration showing a CREB-responsive element (CRE) at the promoter of *Rab3B*. TSS: transcription start site. Bottom, ChIP assays performed using anti-CREB antibody or control IgG. ^*^P < 0.05

Next, we validated the effect of LKB1 on the expression of Rab3B in NSCLC cells. Compared with the NSCLC cell lines (H1299 and H1792) harboring wild-type *LKB1*, *LKB1*-mutated NSCLC cell lines (A549, H460, and H1944) had increased levels of Rab3B (Fig. 2A and B). siRNA-mediated depletion of *LKB1* enhanced Rab3B expression in both H1299 and H1792 cells (Fig. 2C–E). In contrast, LKB1 re-expression in *LKB1*-deficient A549 and H1944 cells suppressed the expression of Rab3B (Fig. 2F).

*LKB1* deficiency has been documented to promote CREB-mediated transcriptional activation in lung cancer cells (Zhou et al. 2021). To check whether loss of LKB1 induces the expression of Rab3B via CREB-dependent transcription, we performed *CREB* knockdown experiments. Silencing of *CREB* abrogated the upregulation of the *Rab3B* mRNA level upon *LKB1* depletion (Fig. 2G–I). Using the ALGGEN-PROMO software, the promoter of *Rab3B* is predicted to have a putative binding site (-879 to -872) for CREB (Fig. 2J). To verify the binding of CREB to the promoter of *Rab3B*, we conducted ChIP experiments using anti-CREB antibody. The results confirmed a significant enrichment of CREB at the promoter region of *Rab3B* (Fig. 2J). Taken together, LKB1 loss-induced upregulation of Rab3B depends on CREB activity in NSCLC cells.

### Rab3B promotes lung adenocarcinoma cell growth, migration, and tumorigenesis

**Fig. 3:**
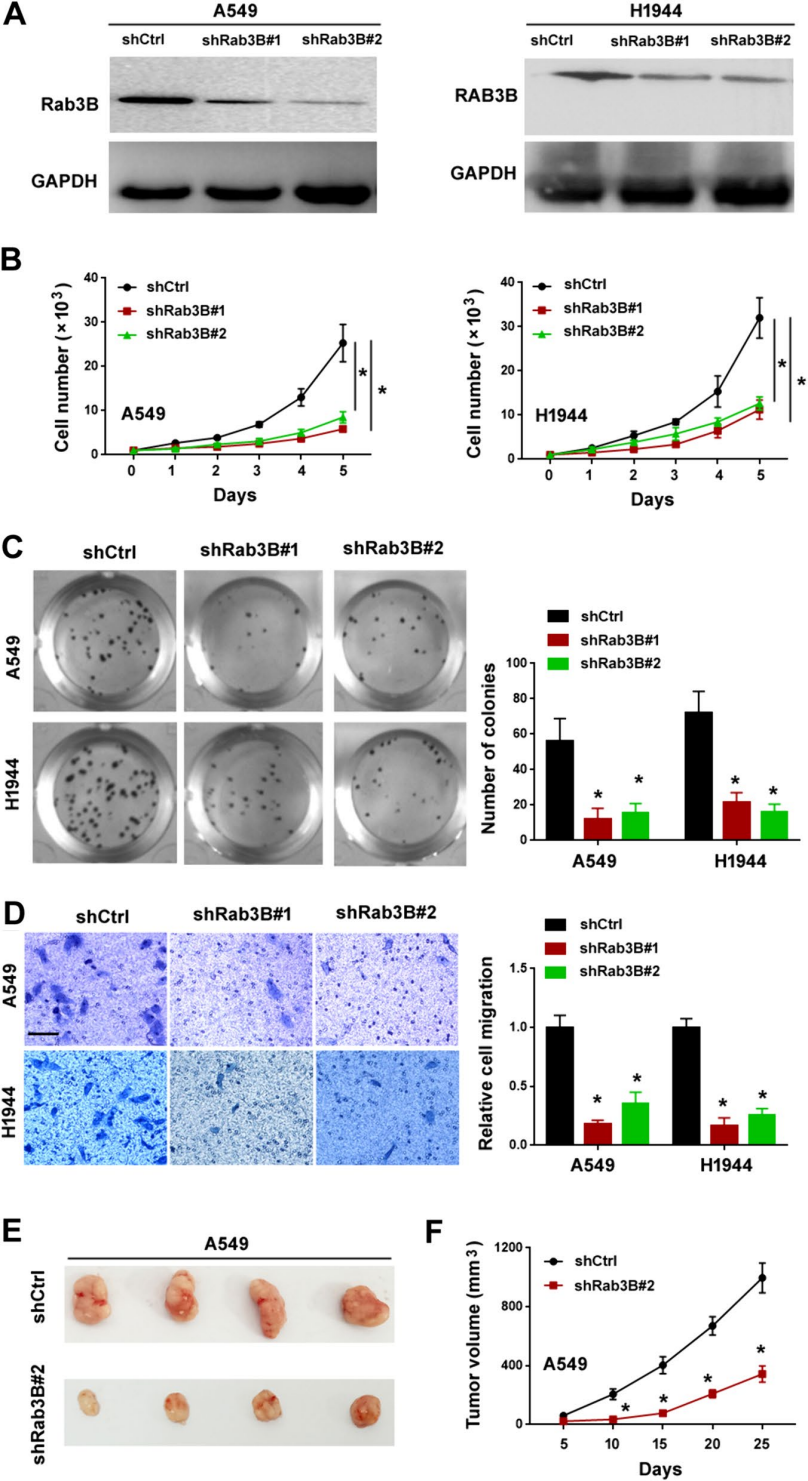
Knockdown of Rab3B suppresses lung adenocarcinoma cell growth, migration, and tumorigenesis. **A** Western blot analysis of Rab3B protein levels in A549 and H1944 cells transfected with indicated shRNAs. **B** Cell proliferation ability determined by cell counting. ^*^P < 0.05. **C** Colony formation assay in A549 and H1944 cells transfected with indicated shRNAs. *Left*, representative images of cell colonies. ^*^P < 0.05. **D** Transwell migration assay in A549 and H1944 cells transfected with indicated shRNAs. *Left*, representative images of migrated cells. Scale bar: 20 μm. ^*^P < 0.05. **E**, **F** Assessment of tumorigenic capacity of A549 cells transfected with indicated shRNAs. **E** Representative xenograft tumors. **F** Tumor growth curves. ^*^P < 0.05

**Fig. 4:**
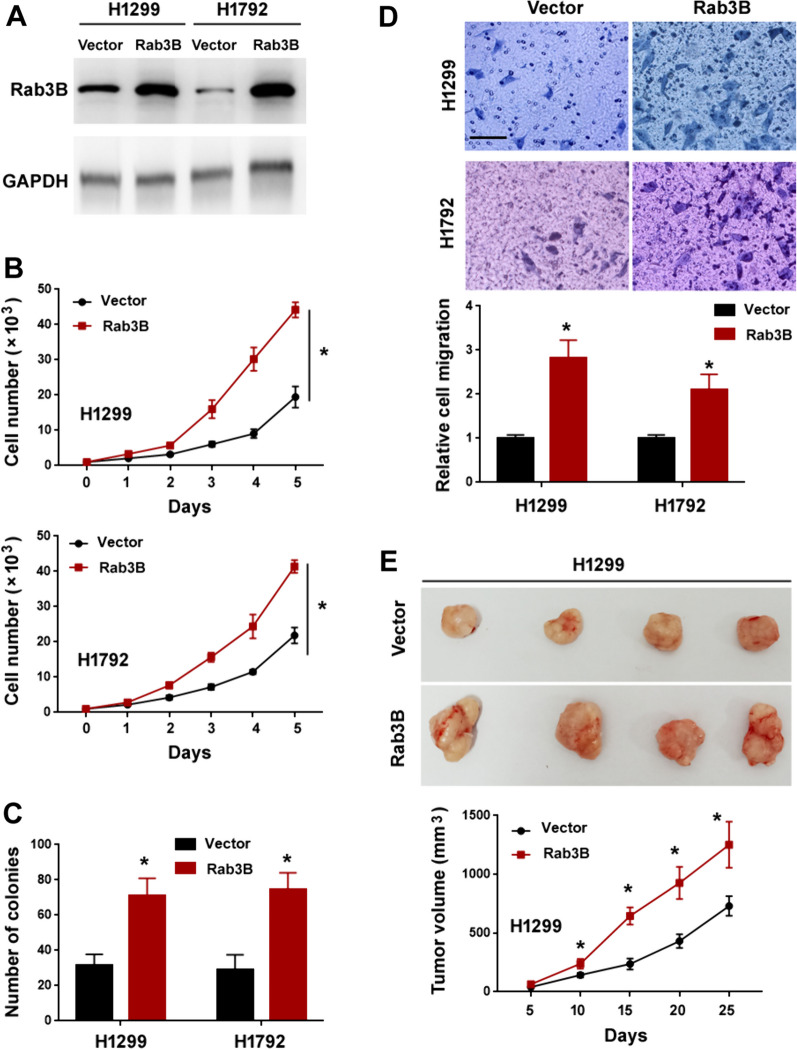
Overexpression of Rab3B promotes lung adenocarcinoma cell growth, migration, and tumorigenesis. **A** Western blot analysis of Rab3B protein levels in H1299 and H1792 cells transfected with indicated constructs. **B** Cell proliferation ability determined by cell counting. ^*^P < 0.05. **C** Colony formation assay. ^*^P < 0.05. **D** Transwell migration assay. Top, representative images of migrated cells. Scale bar: 20 μm. ^*^P < 0.05. **E** Assessment of tumorigenic capacity of H1299 cells transfected with indicated constructs. Top, representative xenograft tumors. ^*^P < 0.05

To investigate the role of Rab3B in NSCLC, we knocked down its expression in A549 and H1944 cells using specific shRNAs (Fig. 3A). Depletion of *Rab3B* resulted in a significant suppression of NSCLC cell proliferation and colony formation (Fig. 3B and C). Transwell migration assay was conducted to evaluate the motility of cancer cells. As illustrated in Fig. 3D, *Rab3B*-depleted NSCLC cells had a reduced migratory capacity relative to control cells. We also checked the effect of depletion of *Rab3B* on NSCLC tumorigenesis in immunodeficient nude mice. We found that *Rab3B* depletion dramatically suppressed the growth of A549 xenograft tumors (Fig. 3E and F). In addition, we performed Rab3B gain-of-function experiments in H1299 and H1792 cells. Overexpression of Rab3B augmented the proliferation, colony formation, and migration of H1299 and H1792 cells (Fig. 4A–D). Moreover, Rab3B overexpression increased the tumorigenic capacity of H1299 cells in nude mice (Fig. 4E). Collectively, these data indicate that Rab3B contributes to the aggressive phenotype of NSCLC cells.

### Rab3B associates with DDX6 and enhances its stability

**Fig. 5:**
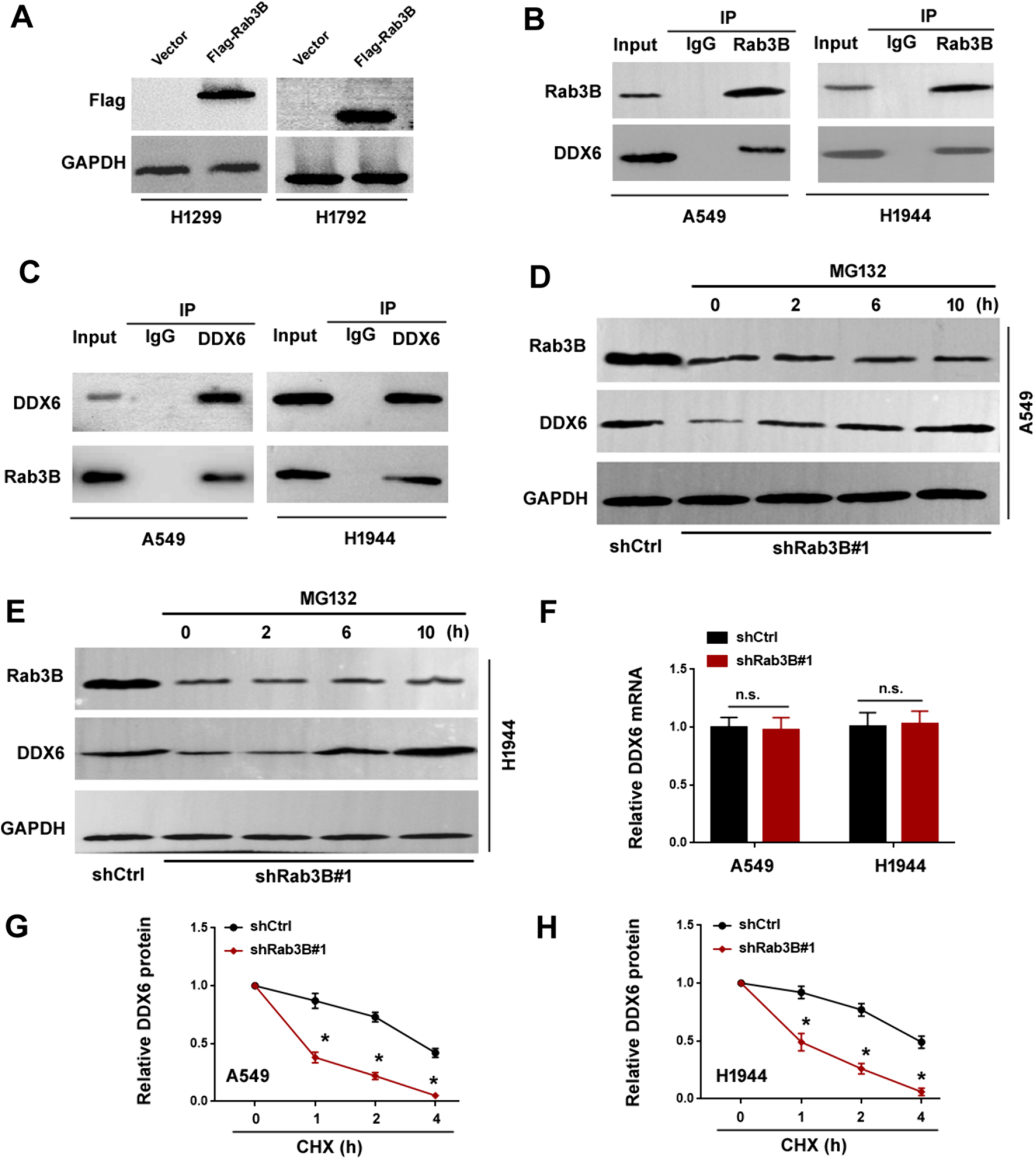
Rab3B associates with DDX6 and enhances its stability. **A** Western blot analysis of Flag-tagged Rab3B in H1299 and H1792 cells. **B** Western blot analysis of Rab3B and DDX6 in Rab3B immunoprecipitates. **C** Western blot analysis of Rab3B and DDX6 in DDX6 immunoprecipitates. **D**, **E** Western blot analysis of Rab3B and DDX6 in A549 **D** and H1944 **E** cells transfected with the *Rab3B*-targeting shRNA and treated with 5 µM MG132 for 2, 6, and 10 h. **F** Measurement of DDX6 mRNA levels. n.s. indicates no significance. **G** A549 and **H** H1944 cells transfected with indicated shRNAs were treated with cycloheximide (CHX) and tested for DDX6 protein levels at the indicated time points. ^*^P < 0.05

To uncover the mechanisms underlying Rab3B-induced aggressive phenotype in NSCLC cells, we sought to identify Rab3B-interacting proteins. Hence, we overexpressed Flag-tagged Rab3B in H1299 and H1792 cells (Fig. 5A) and performed immunoprecipitation experiments and mass spectrometry. DDX6 ranked among the top proteins associated with Rab3B. Since DDX6 plays an oncogenic role in multiple cancers including gastric cancer, colon cancer, and myeloid leukemia (Taniguchi et al. 2018, 2015; Ghashghaei et al. 2022), we focused on the interaction between Rab3B and DDX6. Co-immunoprecipitation assays confirmed the association of endogenous Rab3B with DDX6 in A549 and H1944 cells (Fig. 5B and C). Of note, depletion of *Rab3B* decreased the protein level of DDX6 in A549 and H1944 cells, which was abolished by MG132 treatment for 2–10 h (Fig. 5D and E). In addition, the *DDX6* mRNA abundance remained unchanged when *Rab3B* was depleted (Fig. 5F). We speculated that Rab3B might modulate the stability of DDX6 protein. To address this assumption, we utilized cycloheximide to block protein synthesis. As shown in Fig. 5G and H, in the presence of cycloheximide, the turnover of DDX6 protein was accelerated in *Rab3B*-depleted cells relative to control cells. These data suggest that the association with Rab3B enhances the stability of DDX6 protein.

### DDX6 plays an oncogenic role in lung adenocarcinoma

**Fig. 6:**
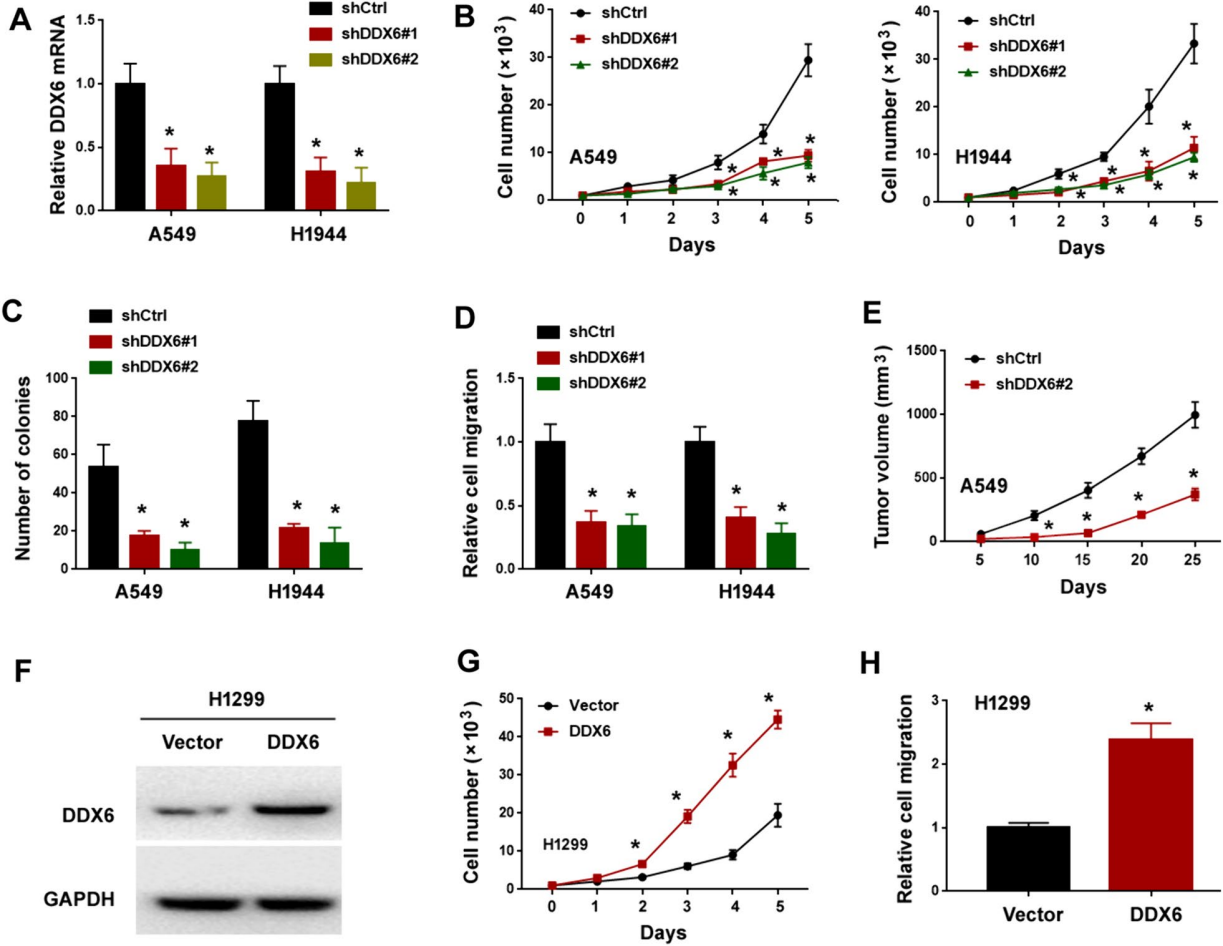
DDX6 plays an oncogenic role in lung adenocarcinoma. **A** Analysis of DDX6 mRNA levels in A549 and H1944 cells transfected with indicated shRNAs. ^*^P < 0.05. **B** Cell proliferation assay in A549 and H1944 cells transfected with indicated shRNAs. ^*^P < 0.05 vs. shCtrl. **C** Colony formation assay in A549 and H1944 cells transfected with indicated shRNAs. ^*^P < 0.05. **D** Transwell migration assay in A549 and H1944 cells transfected with indicated shRNAs. ^*^P < 0.05. **E** Tumorigenesis by A549 transfected with indicated shRNAs in nude mice. ^*^P < 0.05 vs. shCtrl. **F** Overexpression of DDX6 in H1299 cells. **G** Cell proliferation assay in H1299 cells transfected with indicated constructs. ^*^P < 0.05. **H** Transwell migration assay in H1299 cells transfected with indicated constructs. ^*^P < 0.05

To investigate the biological function of DDX6 in NSCLC, we generated stable NSCLC cell lines that expressed *DDX6*-targeting shRNAs. As shown in Fig. 6A, DDX6 expression was dramatically reduced in *DDX6* shRNA-expressing A549 and H1944 cells. When *DDX6* was silenced, the proliferation, colony formation, and migration of A549 and H1944 cells was impaired (Fig. 6B–D). In vivo studies further demonstrated that *DDX6* deficiency hampered the growth of A549 xenograft tumors (Fig. 6E). To substantiate the oncogenic role of DDX6 in NSCLC, we performed DDX6 overexpression studies in H1299 cells. Notably, ectopic expression of DDX6 enhanced the proliferation and migration of H1299 cells (Fig. 6F–H). Overall, these findings demonstrate that DDX6 functions as an oncogene in NSCLC.

### DDX6 mediates Rab3B-induced aggressive phenotype in lung adenocarcinoma

**Fig. 7:**
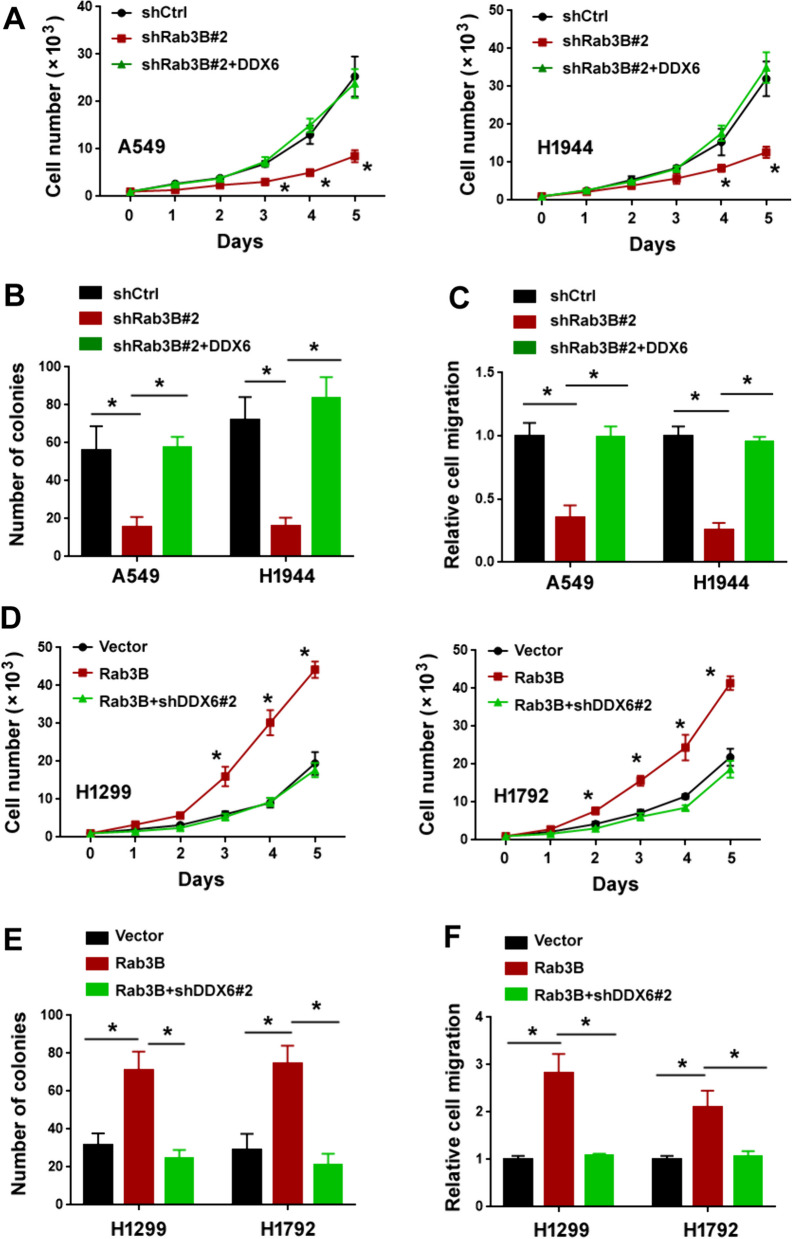
DDX6 mediates Rab3B-induced aggressive phenotype in lung adenocarcinoma. **A** Cell proliferation assay in A549 and H1944 cells transfected with indicated constructs. ^*^P < 0.05 *vs*. shCtrl. **B** Colony formation assay in A549 and H1944 cells transfected with indicated constructs. ^*^P < 0.05. **C** Transwell migration assay in A549 and H1944 cells transfected with indicated constructs. ^*^P < 0.05. **D** Cell proliferation assay in H1299 and H1792 cells transfected with indicated constructs. ^*^P < 0.05 *vs*. Vector. **E** Colony formation assay in H1299 and H1792 cells transfected with indicated constructs. ^*^P < 0.05. **F** Transwell migration assay in H1299 and H1792 cells transfected with indicated constructs. ^*^P < 0.05

As Rab3B and DDX6 play a similar role in driving NSCLC progression, we tested whether DDX6 acts as a partner of Rab3B to mediate Rab3B-induced aggressive phenotype in NSCLC cells. To address this, DDX6 was ectopically overexpressed in Rab3B-depleted NSCLC cells. Overexpression of DDX6 partially rescued the aggressive phenotype in *Rab3B*-depleted A549 and H1944 cells by increasing cell proliferation, colony formation, and migration (Fig. 7A–C). To confirm the importance of DDX6 in the aggressive phenotype mediated by Rab3B, we knocked down *DDX6* in Rab3B-overexpressing H1299 and H1792 cells. Notably, depletion of *DDX6* reversed the effects of overexpression of Rab3B on NSCLC cells by attenuating cell proliferation, colony formation, and migration (Fig. 7D–F). Collectively, these results indicate that Rab3B exerts its oncogenic effects on NSCLC cells via a DDX6-dependent mechanism.

## Discussion

In this article, we reveal the induction of Rab3B in lung adenocarcinoma cells upon LKB1 deficiency. Rab3B upregulation is associated with poor prognosis and promotes aggressive phenotype in lung adenocarcinoma. We also clarify how Rab3B fuels lung adenocarcinoma progression. Specifically, Rab3B interacts with and potentiates the stability of DDX6, which endows cancer cells with more aggressiveness. Our work highlights the importance of the Rab3B-DDX6 axis in lung adenocarcinoma progression.

Several previous studies have reported the involvement of Rab3B in hepatocellular carcinoma, glioma, breast cancer, and prostate cancer (Tsunedomi et al. 2022; Liu et al. 2014; Ye et al. 2014; Tan et al. 2012). Here, we provide first evidence for the oncogenic function of Rab3B in lung adenocarcinoma. Our data show that Rab3B expression is enhanced in lung adenocarcinoma relative to adjacent normal tissues. Elevated Rab3B expression is associated with lymph node metastasis, advanced tumor stage, and shorter overall survival in patient with lung adenocarcinoma. Dysregulation of Rab3B may be a consequence of *LKB1* gene inactivation, as we found that *LKB1*-mutated NSCLC cell lines express higher levels of Rab3B than NSCLC cells carrying wild-type *LKB1*. Moreover, knockdown of LKB1 increases and re-expression of LKB1 suppresses Rab3B expression in NSCLC cells, suggesting the negative regulation of Rab3B by LKB1. Activation of CREB has been suggested to be involved in LKB1-dependent regulation of gene expression (Zhou et al. 2021). Bioinformatic analysis shows that the promoter of *Rab3B* harbors a putative binding site for CREB. ChIP assays validate that CREB protein is enriched at the promoter of *Rab3B* in lung cancer cells. Most importantly, knockdown of *CREB* almost completely blocks the induction of *Rab3B* upon *LKB1* silencing. These results collectively indicate that LKB1 loss enhances the expression through a CREB-dependent mechanism.

Rab3B can regulate breast cancer cell proliferation and invasion (Ye et al. 2014) and prostate cancer cell survival (Tan et al. 2012). Consistently, our data demonstrate that Rab3B can promote the proliferation, colony formation, and migration of lung adenocarcinoma cells. In vivo tumorigenic studies further confirm the tumor-promoting ability of Rab3B. Given the induction of Rab3B upon LKB1 loss, we suggest that Rab3B may function as an oncogenic driver in *LKB1*-deficient cancers.

Rab3B has been shown to exert its biological function through interaction with other proteins (Nishimura et al. 2008; van IJzendoorn et al. 2002; Hu et al. 2023). For example, Rab3B can bind to the polymeric immunoglobulin receptor and regulate transcytosis in epithelial cells (van IJzendoorn et al. 2002). In this study, we show that there is an interaction between Rab3B and DDX6 in lung adenocarcinoma cells. Rab3B is implicated in the stabilization of DDX6 protein, as DDX6 protein turnover is accelerated in the absence of Rab3B. Previous studies have revealed the oncogenic role of DDX6 in gastric cancer, colorectal cancer, and glioblastoma (Taniguchi et al. 2018; Nakagawa et al. 1999; Cho et al. 2016). Given the interaction between Rab3B and DDX6, we speculated that DDX6 might be involved in lung adenocarcinoma progression. Interestingly, we demonstrate that DDX6-depleted lung adenocarcinoma cells recapitulate the phenotype of Rab3B-depleted counterparts. Specifically, depletion of DDX6 blocks the proliferation, colony formation, and migration of lung adenocarcinoma cells in vitro, as well as tumorigenesis in vivo. In contrast, overexpression of DDX6 enhances the aggressiveness of lung adenocarcinoma cells. Most importantly, ectopic expression of DDX6 can partially rescue the proliferation, colony formation, and migration potential in Rab3B-depleted lung adenocarcinoma cells. Rab3B-meidated aggressive phenotype in lung adenocarcinoma cells is impaired when DDX6 is silenced. These results indicate that DDX6 mediates the tumor-promoting effects of Rab3B on lung adenocarcinoma cells.

DDX6 as a RNA-binding protein plays an important role in post-transcriptional regulation of gene expression. It has been documented that DDX6 can repress the activation of interferon-stimulated genes through regulation of RNA processing (Lumb et al. 2017). DDX6 can also repress gene expression through the microRNA-mediated silencing pathway (Kim et al. 2022; Rouya et al. 2014). In mammalian progenitor cells, DDX6 can bind to the 3'-untranslated regions (UTRs) of *CDK1* and *EZH2* to enhance their translation and also facilitate the degradation of *KLF4* transcript through interaction with its 5'-UTR (Wang et al. 2015). In gastric cancer cells, DDX6 has been found to interact with c-Myc mRNA and thus enhance the mRNA and protein expression of c-Myc (Taniguchi et al. 2018). These studies provide an explanation for the regulation of downstream genes by DDX6 to exert its biological effects. However, the mechanism by which DDX6 promotes lung adenocarcinoma progression needs to be clarified in future work.

## Conclusion

Taken together, our work indicates that Rab3B is upregulated in *LKB1*-deficient lung adenocarcinoma cells and drives lung adenocarcinoma progression through interaction with and stabilization of DDX6 protein. Rab3B upregulation is correlated with advanced disease and poor prognosis in patients with lung adenocarcinoma. CREB is required for the upregulation of *Rab3B* upon *LKB1* loss. Therefore, the Rab3B-DDX6 axis may be a potential therapeutic target in LKB1-deficient lung adenocarcinomas.

## Data Availability

The data that support the findings of this study are available from the corresponding author upon reasonable request.
